# Diphenhydramine as a Cause of Drug-Induced Liver Injury

**DOI:** 10.1155/2017/3864236

**Published:** 2017-01-26

**Authors:** Yunseok Namn, Yecheskel Schneider, Isabelle H. Cui, Arun Jesudian

**Affiliations:** ^1^Department of Gastroenterology and Hepatology, Weill Cornell Medical College, New York Presbyterian Hospital, New York, NY, USA; ^2^Department of Pathology, Weill Cornell Medical College, New York Presbyterian Hospital, New York, NY, USA

## Abstract

Drug-induced liver injury (DILI) is the most common cause of acute liver failure in the Unites States and accounts for 10% of acute hepatitis cases. We report the only known case of diphenhydramine-induced acute liver injury in the absence of concomitant medications. A 28-year-old man with history of 13/14-chromosomal translocation presented with fevers, vomiting, and jaundice. Aspartate-aminotransferase and alanine-aminotransferase levels peaked above 20,000 IU/L and 5,000 IU/L, respectively. He developed coagulopathy but without altered mental status. Patient reported taking up to 400 mg diphenhydramine nightly, without concomitant acetaminophen, for insomnia. He denied taking other medications, supplements, antibiotics, and herbals. A thorough workup of liver injury ruled out viral hepatitis (including A, B, C, and E), autoimmune, toxic, ischemic, and metabolic etiologies including Wilson's disease. A liver biopsy was consistent with DILI without evidence of iron or copper deposition. Diphenhydramine was determined to be the likely culprit. This is the first reported case of diphenhydramine-induced liver injury without concomitant use of acetaminophen.

## 1. Introduction

Drug-induced liver injury (DILI) is the most common cause of acute liver failure in the Unites States, accounting for nearly 50% of cases and 10% of acute hepatitis cases [1–3]. Other etiologies of acute liver injury include viral, autoimmune, metabolic, and ischemic etiologies. When acute liver injury progresses to fulminant hepatic failure, it is associated with high mortality [4, 5]. Therefore, prompt recognition of the underlying etiology and initiation of treatment are of paramount importance. Medications such as acetaminophen, antiepileptics, and antibiotics are well known causes of DILI; however, thousands of drugs, herbs, and toxins can cause idiosyncratic liver injury. We report the first case of diphenhydramine-induced liver injury without concomitant use of acetaminophen.

## 2. Case

A 28-year-old man with history of chromosomal translocation 13/14 and no prior liver disease presented to the hospital with complaint of fevers, nonbloody emesis, and dark urine. History revealed ingestion of diphenhydramine 400 mg nightly, taken for insomnia, over the previous 4 months; the medication was inspected in the hospital and confirmed not to contain acetaminophen. He denied use of herbal compounds, supplements, teas, and any other medication. The patient endorsed rare alcohol consumption and rare intranasal cocaine use in the distant past. Family history was notable for possible statin-induced liver disease and hereditary angioedema.

**Figure 1:**
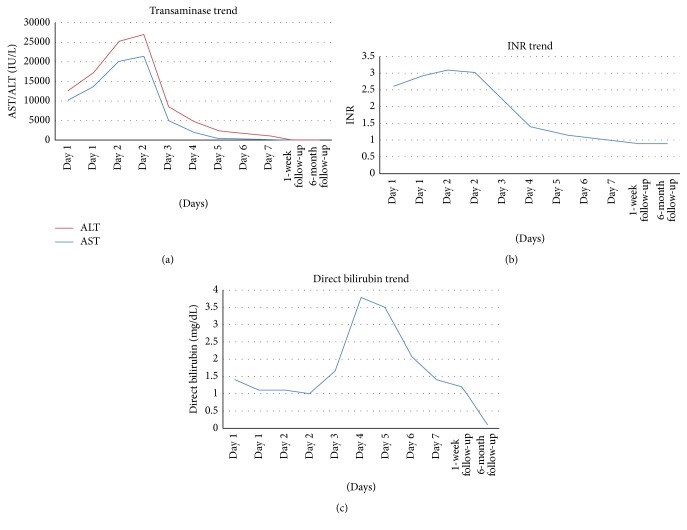
Linear graphs representing the trend of transaminases, INR, and direct bilirubin throughout patient's hospital course and outpatient follow-up.

**Figure 2:**
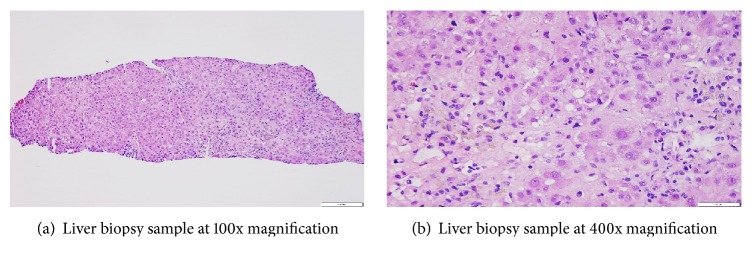
On Hematoxylin and Eosin stain (100x and 400x), there is centrilobular and bridging necrosis accompanied by portal and lobular inflammation including eosinophils and plasma cells. There is evidence of cholestasis and bile duct injury is noted in some portal tracts. Hepatocellular injury, that is, hepatocyte ballooning degeneration, is also present. Fibrosis is absent on Trichrome staining. There is no evidence of steatosis, glycogenic nuclei, Mallory's hyaline, or copper deposition. Overall, these findings favor a diagnosis of drug-induced liver injury.

Vital signs on presentation were within normal limits. Physical exam revealed icteric conjunctiva, a 15 cm liver palpable 3 cm below the right costal margin, nonpalpable spleen, normal mentation, and neurologic exam without asterixis. Initial laboratory studies revealed aspartate aminotransferase (AST) 10,425 IU/L, alanine aminotransferase (ALT) 2,471 IU/L, alkaline phosphatase (ALP) 65 IU/L, total bilirubin 2.7 mg/dL (direct 1.4 mg/dL), INR 2.6, and a hemoglobin level of 12.7 g/dL; the remainder of the laboratory studies were normal, including creatinine, blood glucose, and lactate. Hepatology and toxicology consultations were requested, and empiric intravenous N-acetylcysteine (NAC) was initiated. By day two, his transaminases and coagulopathy worsened, with AST 20,176 IU/L, ALT 5,076 IU/L, and INR 3.1 (Figures 1(a), 1(b), and 1(c)). The patient did not exhibit signs of hepatic encephalopathy, but was transferred to the intensive care unit for closer monitoring. By the third day of NAC treatment, transaminases started to improve. A complete evaluation for causes of acute liver injury, including viral hepatitis (hepatitis A, B, C, and E), workup for other viruses (including EBV, CMV, HHV-6, and HIV), autoimmune workup (ANA, ASMA, AMA, A1AT, IgG, and LTK Ab), metabolic testing (ceruloplasmin, 24-hour urine copper, ophthalmologic examination for Keyser-Fleischer rings, MRI brain to evaluate for lenticular degeneration, and iron studies), urine toxicology, and SPEP/UPEP, was all normal and did not reveal an alternative etiology of the patient's acute liver injury. Abdominal ultrasound demonstrated a 17.4 cm liver with smooth contour and normal echogenicity with patent hepatic and portal vasculature. He underwent a transjugular biopsy which revealed centrilobular and bridging necrosis accompanied by portal and lobular inflammation, favoring drug-induced liver injury (Figures 2(a) and 2(b)). Rhodanine stain for copper and quantitative copper analysis were both negative, ruling out Wilson's disease. Iron staining was not consistent with hemochromatosis. The patient ultimately recovered completely from acute liver injury and was discharged home. Upon the 6-month outpatient follow-up, patient was asymptomatic and reported feeling well, and repeat liver function tests did not show any evidence of liver injury.

## 3. Discussion

Acute liver injury can be attributed to a wide array of causes, with viral and drug etiologies being the most common in adults. In the United States, acetaminophen toxicity and idiosyncratic drugs reaction are the leading causes of acute liver failure while viral hepatitis is the leading cause in Asia and Europe [4, 5].

Drug-induced liver injury remains one of the most challenging diagnoses to make but must be considered in the differential diagnosis of any liver injury [6, 7]. Patients should undergo careful evaluation to exclude other causes of liver disease, including viral hepatitides (hepatitis A, B, C, and E), EBV, CMV, HHV-6, autoimmune hepatitis, and Wilson's disease if age is less than 40 [8, 9]. Offending agents can include prescription and over-the-counter medications, such as antibiotics (nitrofurantoin, isoniazid, and trimethoprim/sulfamethoxazole), immunomodulatory agents (such as infliximab), analgesics (especially those that contain acetaminophen), antineoplastic agents, antiepileptics, and statins [10, 11]. Additionally, DILI can result from herbal ingestions or dietary supplements [12, 13]. Manifestations of liver injury can range from asymptomatic transaminase elevation and/or jaundice (depending on a hepatocellular or cholestatic pattern of injury, resp.) to development of acute liver failure marked by encephalopathy and synthetic dysfunction [13, 14].

Drug-induced liver injury can be divided into two classes of medication-related hepatotoxicity: intrinsic and idiosyncratic. The former, which includes acetaminophen, is often dose-dependent with predictable liver injury at hepatotoxic doses [11, 15]. Idiosyncratic DILI is not dose-dependent and thus is harder to predict. It is thought to mainly affect individuals with underlying susceptibility or predisposition and therefore, may have variable latency [11, 16].

In the presented case, multiple etiologies for the liver injury were considered and excluded. The liver biopsy was consistent with DILI and after ascertainment of an exhaustive mediation history diphenhydramine was identified as the only possible culprit. Although there are reports of diphenhydramine and acetaminophen causing liver injury when ingested together, there are no prior cases in the literature of diphenhydramine alone causing DILI. Although considered to have minimal to no hepatotoxic effects, this case suggests that diphenhydramine may be hepatotoxic at high doses with chronic use (as the patient had been taking at least 400 mg per day for over four months). It has been reported that compounds with more than 50% hepatic metabolism given at doses higher than 50 mg/day were at highest risk of hepatotoxicity [13, 17]. Diphenhydramine undergoes extensive first-pass metabolism, whereby 50–60% of ingested medication is metabolized by the liver before reaching the systemic circulation. Nearly all the available drug is metabolized by the liver within 24–48 hours, thus increasing risk for liver injury. It is unclear if a predisposition for hepatotoxicity was present in this patient given his history of a balanced chromosomal translocation. However, certain genetic predispositions have been noted to play a role in risk for DILI. For example, the HLA-1 and HLA-II genotypes have been shown to confer an increased susceptibility to DILI for patients taking amoxicillin-clavulanate [7, 17, 18].

In summary, we present a case of diphenhydramine-induced liver injury, without the concomitant use of acetaminophen. Although not previously thought to cause liver injury, this case highlights that any medication metabolized by the liver has potential to lead to liver injury when ingested at high enough doses and for long enough duration in susceptible individuals.
